# A 12-month randomized controlled trial to assess the efficacy of revitalization of retreated mature incisors with periapical radiolucency in adolescents

**DOI:** 10.1038/s41598-024-66305-5

**Published:** 2024-07-16

**Authors:** Ahmad Abdel Hamid Elheeny, Osama Seif-Elnasr Hussien, Mahmoud Ahmed Abdelmotelb, Yassmin Mohamed ElMakawi, Norhan Khaled Omar Wahba

**Affiliations:** 1 Paediatric and Community Dentistry, Faculty of Dentistry, Minia University, Ard Shalaby, El Minia, Minya, 61519 Egypt; 2 Paediatric Dentistry, Faculty of Dentistry, Minia University, Minya, Egypt; 3 Paediatric and Community Dentistry, Faculty of Oral and Dental Medicine, Nahda University, New Bani Suef, Egypt

**Keywords:** Paediatric dentistry, Health care, Dentistry

## Abstract

The use of regenrative endodontics is restoring the health status of the root canals of retreated mature teeth is a novel approach. Therefore, the current trial aimed to compare the effectiveness of regenerative endodontic procedures (REPs) to non-surgical root canal retreatment (NS-RCR) in reducing periapical radiolucency over one year for the retreatment of mature incisors with periapical periodontitis. The secondary purpose was to assess clinical success and regain pulp sensibility. A parallel randomized controlled trial, 66 mature incisors with periapical radiolucencies were randomly divided into two equal groups and retreated with either REPs or NS-RCR. At baseline and after 6 and 12 months, teeth were assessed clinically and radiographically using a periapical index (PAI). The Mann–Whitney test was used to analyze nonparametric PAI scores. The Electric pulp test readings were analyzed using the repeated measure analysis of variance (ANOVA). Over the follow-up intervals, there was no significant intergroup difference in the PAI medians, the majority of the teeth displayed a reduction in periapical radiolucency. At the end of the follow-up period, the clinical successes for the REP and NS-RCR groups were 93.9% and 97%, respectively (*p* = 0.555). Positive pulp sensibility was recorded in 54.54% of cases in the REPs after 12 months. Both approaches showed a comparable diminishing of periapical radiolucencies and equivalent clinical results. A conventional, non-surgical endodontic retreatment may not always be necessary.

## Introduction

Although non-surgical root canal retreatment (NS-RCR) is the standard treatment for failed primary endodontic therapy, its failure rate ranges from 23 to 30%^1,2^. Similar to primary endodontic therapy, NS-RCR can’t totally prevent bacterial recolonization even after chemomechanical instrumentation^3,4^. Additional disadvantages of NS-RCR include loss of viability, deterioration of the remaining tooth structure, and the absence of a natural innate immune defense mechanism^5,6^.

A paradigm shift toward an alternate endodontic treatment philosophy has been recognised with the introduction of the regenerative endodontic concept. Inducing blood clot (BC) formation in the disinfected root canal space is one of the regenerative endodontic procedures (REPs) techniques^7^. The REP satisfies the three main integrated components of tissue engineering^6^. The BC serves as a scaffold to support stem cells' migration and proliferation from the region of the apical papilla into the root canal space. It delivers the growth factors necessary for the development and differentiation of stem cells^8^. Compared to the NS-RCR, the REPs aim to reconstruct blood supplies, maximise the healing process, allow root neurogenesis and subsequent increase in root thickness, and permit the organisation of viable tissues into the root canal space of teeth with periapical pathosis^4,9^.

Although the histological findings of immature teeth with apical periodontitis confirmed that the dental papilla, which is the fundamental reservoir for mesenchymal stem cells (SCAPs), is essential for root maturogenesis and remains vital after periapical infection^10,11^, these cells are not present in the dental papilla of mature teeth with apical periodontitis. The human and animal studies revealed that the nature of regenrated tissues inside the pulp space was of periodontal origin, with the formation of cementum and bone-like tissues^10^.

In secondary infections, *E. faecalis* is the dominant isolated pathon, with a prevalence of up to 70%. *E. faecalis* is highly resistant to various disinfectants and antimicrobes, as well as its ability to penetrate into the dentinal tubules, diluting the action of antispetics^12^. Complete suppression of *E. faecalis* depends on the concentration of NaOCl, intracanal medication type and duration of application, and irrigant activation use^12^. Calcium hydroxide (CH) is one of the commonly used and recommended intracanal temporary dressing materials that proved to be efficient in reducing bacteria load on the root canal walls, while being less effective against those deep in the dentinal tubules^13^. The irrigation of 1.5% NaOCl during REPs is shown to be effective against *E. faecalis* in a 3-week old bacterial biofilm^14^.

The majority of studies concentrate on the clinical application of REPs in the management of necrotic, immature young permanent teeth. The pooled data from two systematic reviews and meta-analyses showed high survival rates of immature permanent teeth treated with REPs^15,16^. Although there are currently no defined standards that suggest using REPs as an approved method of treating mature permanent teeth with necrotic pulp tissues, the findings of previous studies were unable to offer solid proof in favour of the efficacy of revitalisation in either immature or mature permanent teeth with apical periodontitis^6,17–19^.

The favorable results of necrotic mature teeth with apical perioidontitis in previous studies encouraged us to conduct this investigation^6,20,21^. No prior prospective study has examined the efficacy of REPs in the retreament of necrotic, mature permanent teeth identified as having periapical periodontitis. The current experiment had been conducted to utilize the benefits of REPs in treating nectrotic mature permanent teeth by reorganizing the innate immune system, restoring pulp vascularity, and reducing the risk of reinfection^4^. Another privilege of the REPs could increase these teeth' fracture resistance compared to NS-RCR^20^. Therefore, the primary aim of the study was to assess the effectiveness of REPs in reducing periapical radiolucency over one year for the retreatment of mature incisors with periapical periodontitis compared to NS-RCR. The secondary purpose was to assess clinical success and regain of pulp sensibility.

## Materials and methods

### Ethical standards

The trial was reviewed and approved by the Researcch Ethics Committee, Faculty of Dentistry, Cairo University (reference no. 40922). The study started in December 2021 and was completed in October 2023 after registration at ClinicalTrials.gov “reference no. NCT05168553 (23/12/2021)”. A signed informed consent was obtained from each parent/caregiver before starting the trial. An informed consent was obtained from each patient aged 16 years or older. All procedures in studies involving human participants were performed in accordance with the ethical standards of the institutional and/or national research committee and with the 1964 Helsinki Declaration and its later amendments or comparable ethical standards.

### Sampling

Since no previous trial had addressed the diminishing in periapical radiolucency of retreated mature permanent teeth with REPs compared to NS-RCR, a pilot study that included 20 teeth (10 teeth per group) was conducted. Hedges’s *g-*a corrected effect size-was calculated for the difference between the two group means was calculated^22^ where the means and standard deviations (SD) of the periapical index (PAI) scores of intervention and control groups were 3.44mm ± 0.65 and 3.87mm ± 0.95 respectively. The estimated unbiased effect size (Hedges’s *g*_*s*_) was 0.658. Analyses were performed at an alpha level of significance of 0.05 and a study power of 0.80. A total of 66 teeth was calculated using G*Power 3.1.9.4 software after adding 10% to consider the possible drop-off.

### Randomization, allocation and blinding

Teeth were distributed at random using a permuted block randomization technique with a block size of four. An independent investigator organised the randomization procedures using computer-generated software. A total of 66 identical, properly sealed, opaque envelopes were created before the clinical procedures began. Envelopes contained identical double-folded paper sheets wrapped within alumnium foil. According to the printed code on the paper sheet indicating the retreatment technique, each envelope was placed in one of the two identical containers. Two envelopes were chosen from each pile and shuffled by an impartial nurse who was unaware of the trial's purpose, nature, allocation, and treatment codes. The patient's legal guardian randomly picked one envelope^23^. The participants were divided into two parallel arms, one was treated with REPs using the triggered BC approach (intervention), and the other (control) was treated with NS-RCR.

### Eligibility standards

#### Inclusion standards

- Under-18-year-old healthy adolescents with mature permanent single-rooted previously traumatized incisors with chronic apical abscess or asymptomatic apical periodontitis that ere indicated for retreatment.
- Only teeth with a periapical index (PAI) score ≥ 3.

#### Exclusion standards

- Teeth with previous attempts for endodontic retreatment were excluded.
- Teeth with periodontal problems, non-restorable crowns, teeth that needed post-placement, and non-restorable crowns.
- Teeth with previous endodontic treatment and a history of severe luxative injuries
- Tooth candidates for endodontic surgery including (1) getting a biopsy, (2) removing intracanal broken files, (3) persistent periapical lesions standing for a long time, (4) radicular perforation, (5) root fracture, (6) obstructed root canals, (7) being unable to remove all of the old gutta-percha, or (8) having material protrude beyond the apex with persistent apical lesions for a long duration.

### Clinical procedures

#### Intervention group

A two-appointment approach was considered for both groups. At the first visit, the tooth was anesthesized with one cartridge (1.7 mL) of articaine hydrochloride 4% and epinephrine 1:100,000 (Septocaine^®^, SEPTODONT Ltd. Paris, France). A rubber dam clamp was placed after confirming the lip numbness. The old restoration was removed from a previously accessed cavity. The heat and Hedstrom file was used to remove the gutta-percha. The gutta-percha was softened using a hot plugger, and then a suitable-sized H-file (MANI Inc., Tochigi, Japan) was screwed into and pulled out of the gutta-percha. No chemical solvents were used, and a periapical radiograph was used to make sure there were no gutta-percha residues^24^. The working length was determined, using an electronic apex locator (DentaPort ZX, J. Morita Corp., Kyoto, Japan). Using a 30-gauge side-vented needle (Endo-Top^®^; PPH CERKAMED, Kwiatkowskiego, Poland), the root canal was cleaned with a 1.5% NaOCl irrigation solution for 20 mL over 5 min. The irrigation needle was adjusted to its working length at 1 mm from the root apex. The root canal was then filled for 5 min with 20 mL of 17% ethylenediaminetetraacetic acid (Prevest, DenPro. Jammu, India). Alternate irrigation with NaOCl and EDTA facilitated the debonding of the remaining filling material. Finally, 20 mL of 0.9% saline solution was used to cleanse the root canal. After the root canal had dried out, calcium hydroxide (CH) (UltraCal, Ultradent, Utah, USA) was inserted as an intracanal medication. (Equia Fil™, GC Corp., Tokyo, Japan) a glass ionomer was used to close the access^6^.

After two weeks, the second appointment was scheduled. The tooth was isolated with a rubber dam after a 3% mepivacaine plain local anaesthetic injection (Scandonest^®^, Septodont, Saint-Maur-des-Fosses, France) was applied. Coronal restoration and CH intracanal dressing were removed using 5 mL of 0.9% saline. Final irrigation with 5 mL of 0.9% saline and 20 mL of 17% EDTA that was passively activated with an ultrasonic noncutting tool for one minute was carried out. Suitable-sized paper points were introduced to dry the root canals.

To trigger intracanal BC formation, a sterile K-file size #35 (MANI Inc., Tochigi, Japan) was passed 2 mm beyond the apical foramen. Bleeding was extended to the cementoenamel junction (CEJ). The cotton pellet was left for 5 min until clotting occurred. As instructed by the manufacturer, Biodentine (Biodentine^®^, Septodont, Saint-Maur-des-Fossés, France) was packed against a collagen resorpable matrix (Collacone^®^, Botiss Biomaterials, Berlin, Germany) that was placed over the created BC^19^. Before restoring the coronal portion, a radiograph was taken for qualitative assessment. A base of light-cured resin-modified glass ionomer restorative material (Riva light cure, SDI, Bayswater, Australia) and composite resin was used to seal the access (Tetric N Ceram Bulk Fill, Ivoclar, Vivadent AG, Schaan, Liechtenstein).

#### Control group

Similar to the intervention group, the old gutta-percha was removed. To evaluate the working length, a suitable-sized initial file engaged in the root canal was inserted with the use of a periapical radiograph and electronic apex locator (DentaPort ZX Apex Locator, J. Morita Corp., Kyoto, Japan). A minimum of 3 successive manual K-files (MANI Inc., Tochigi, Japan) were used for mechanical instrumentation. The access cavity temporization procedure, dryness protocol, and CH intracanal administration were also followed. Patients were recalled two weeks later. After confirming that signs and symptoms were relieved, root canals were filled with gutta-percha (Dia-Pro W™, Diadent^®^, Chungcheongbuk-do, Korea) and resin-based root canal sealer (ADSEAL, Meta Biomed Co., Chungcheongbuk-do, Korea). The access cavity was sealed, just like the REPs group.

Postoperative radiographs were taken in both groups to evaluate the obturation’s quality and provide a baseline for future comparison. In the control group, teeth with overextended fillings were excluded from the analysis.

### Outcomes assessment

#### Radiographic reduction of the periapical radiolucency

Radiographic assessments were performed at baseline (T0), after 6 months (T1) and after 12 months (T2). A long cone "paralleling technique" with a receptor holder was used to assess the changes in periapical radiolucency uniformly after mounting a size 2 periapical film (SKYDENT E, Skýcov, Slovakia) to a silicone biting stick^6,25^.

The PAI classification system was used to monitor the dimensional changes in the periapical radiolucency^26^. The PAI method includes 5 scores: "1" indicates normal periapical structure, "2" and "3" indicate minor alterations in bone structure and some mineral loss, respectively, and "4" and "5" indicate severe periodontitis with aggravating characteristics. Scores of "1" and "2" indicated healthy periodontal structures, whereas scores of "3", "4" and "5" denoted sick periodontal structures.

#### Clinical success and pulp sensibility test

After 6 months (T1) and after 12 months (T2), if any of the following characteristics were observed during an examination: (i) pain on percussion, (ii) sinus tract oedema, (iii) abnormal tooth movement, or both—clinical failure would result. At T1 and T2, the electric pulp tester (EPT) (Pulp Tester DY 310, Denjoy Dental Co.) was used to assess the pulp sensations in the intervention group. The tooth was dried and isolated, then a small amount of fluoridated tooth was applied. The patient's lip was attached to the metal clip. For comparison, baseline reading was established through testing the reading of the adjacent tooth.The average of the three EPT measurements taken at 5-min intervals was calculated.

#### Standardization and calibration

A single expert in pediatric dentistry with seven years of experience in RES performed all clinical procedures. For calibration, 20 reference x-rays were independently assessed by each examiner. At the baseline (T0) and follow-up intervals (T1 and T2), the radiographic examination was carried out independently by two endodontic professionals. The inter-examiner reliability and the intra-examiner reliability were tested using Cohen's Kappa statistics (κ) and intra-class correlation coefficient (ICC). The assessors were blinded to the allocation group during the clinical evaluation. While masking the nature of treatment was impossible during the radiographic assessment.

### Statistical analysis

Data were analysed using IBM SPSS® Statistics version 21. Data were checked for the normality of the distribution of PAI scores using the Kolmogorov–Smirnov, and Shapiro–Wilk tests. To compare the PAI median values between both groups, the Mann–Whitney test was used. Within each group, the change of PAI medians over the follow-up intervals was tested using Wilcoxon signed rank tests (T0 versus T1, T0 versus T2 and T1 versus T2). Using the repeated measure analysis of variance, the normally distributed EPT values at the T1 and T2 follow-up points in the REPs group were examined (ANOVA). The alpha level of significance was set at 0.05 at 95% CI.

### Research involving human participants and/or animals

All procedures in studies involving human participants were performed in accordance with the ethical standards of the institutional and/or national research committee and with the 1964 Helsinki Declaration and its later amendments or comparable ethical standards.

### Informed consent

Clinical procedures were launched after obtaining written informed consent from each participant's legal guardian. An informed consents were obtained from each patient of 16 years or above.

## Results

**Figure 1:**
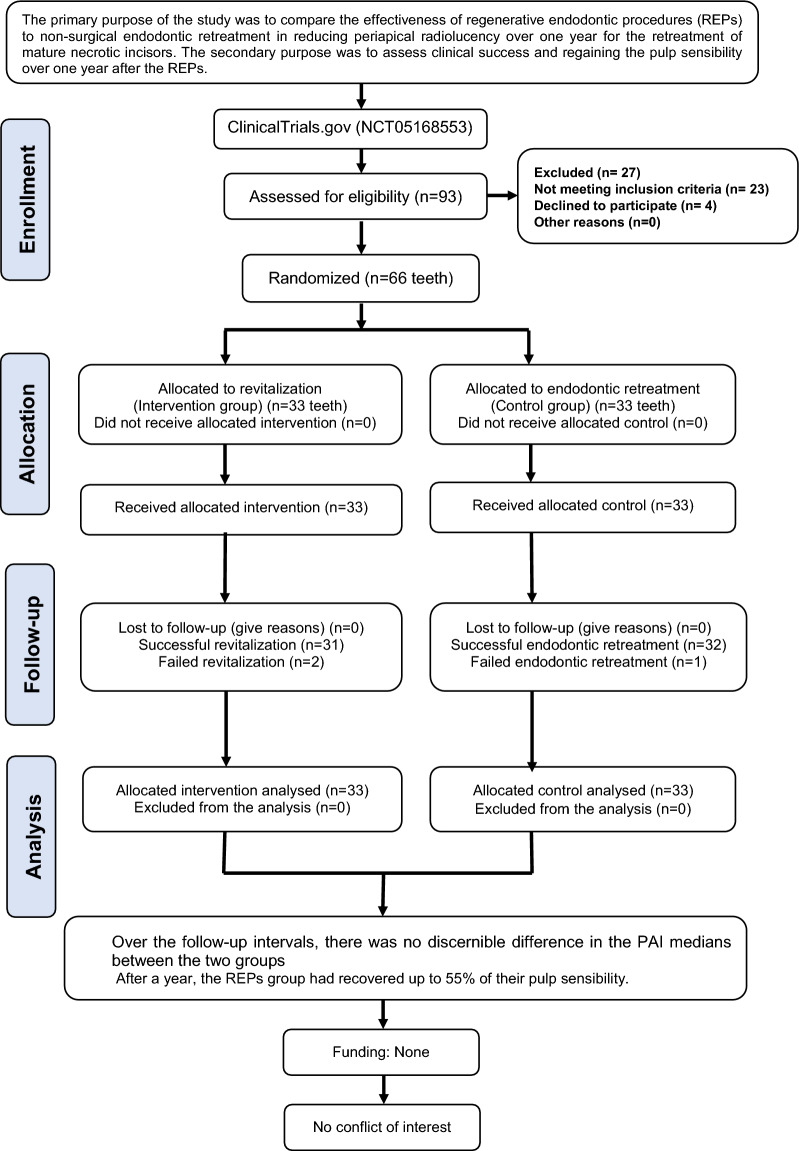
PIRATE flowchart of the study.

Of the 93 teeth assessed for eligibility, 66 teeth were randomly divided into two equal groups (33 teeth per group) (Fig. 1). The age range of the participants was 11 to 17 years, with average ages of 15.29 ± 1.64 and 15.79 ± 1.93 years for the REPs and control groups, respectively. The history of trauma type was mostly a result of complicated crown fractures (66.7% in the NS-RCR group and 60.6% in the REPs group) (*p* = 0.807). The clinical success rates of REPs and NS-RCRs were 93.9% (n = 31) and 97% (n = 32), respectively, at T1 and T2. One tooth in the REPs group displayed the sinus tract reemerging at T1. No statistically significant difference between the two groups was detected (*p* = 0.555) (Table 1). Excellent inter-examiner and intra-examiner reliability were detected. Cohen's Kappa values at T1 and T2 were 0.89 and 0.91, but the ICCs were 0.90, and 0.87, respectively.

**Table 1:** Participant’s baselines, tooth type, clinical success at 6 months (T1) and 12 months (T2).

Variables	NC-RCR group, N (%)	REP group, N (%)	P*
Gender			
Male	22 (66.7)	27 (81.8)	0.159
Female	11 (33.3)	6 (18.2)	
Age (years)			
Mean ± SD	15.29 ± 1.64	15.79 ± 1.93	0.394
Tooth type			
Maxillary central incisors	25 (75.8)	25 (75.8)	0.291
Maxillary lateral incisors	6 (18.2)	5 (15.2)	
Mandibular central incisors	2 (6.1)	3 (9.1)	
Type of trauma			
Complicated crown fracture	22 (66.7)	20 (60.6)	0.807
Uncomplicated crown fracture	5 (15.2)	7 (21.2)	
Subluxation	6 (18.2)	6 (18.2)	
Clinical assessment			
At T1			
Success	32 (97.0)	31 (93.9)	0.555
Failure	1 (3.0)	2 (6.1)	
At T2			
Success	32 (97.0)	31 (93.9)	0.555
Failure	1 (3.0)	2 (6.1)	

*REPs* regenerative endodontic procedures, *SD* standard deviation.

*All variables were compared using a Chi-square test except for the age means that compared using independent sample *t-*test and clinical assessment that compared using Fisher’s Exact test.

**Figure 2:**
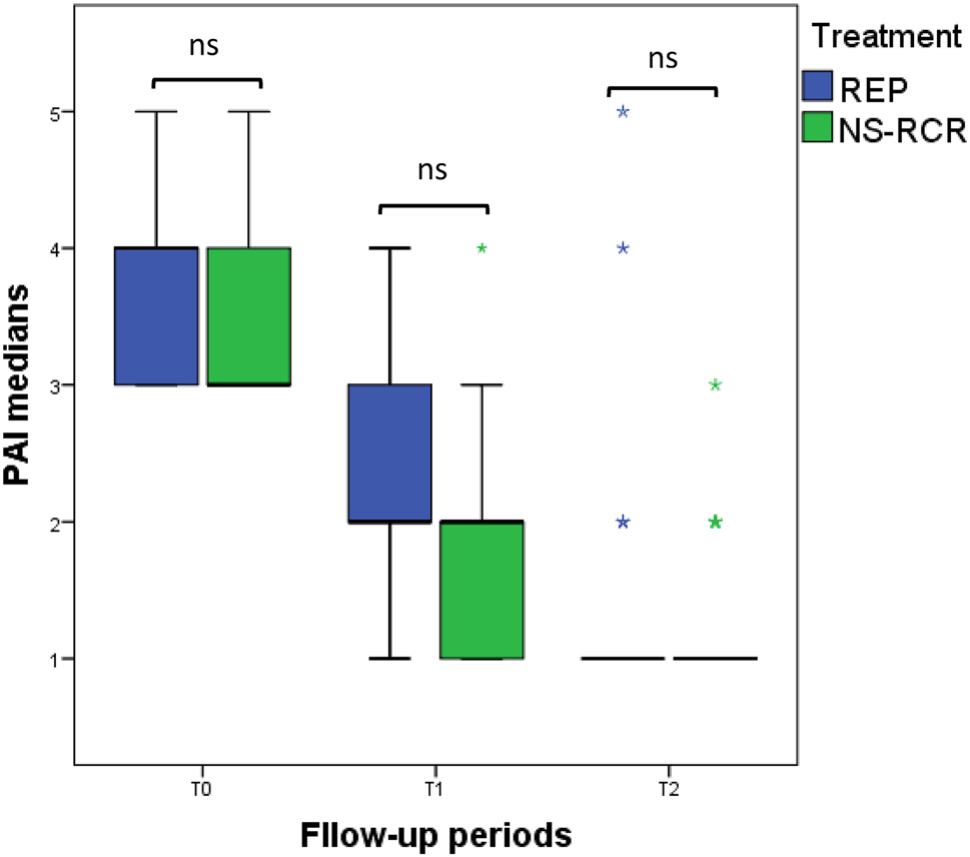
Intergroup comparison of the periapical index (PAI) median scores of the regenerative endodontic procedures (REPs) (intervention group) versus non-surgical endodontic retreatment (control group) at the follow-up intervals (T0: preoperative, T1: after 6 months, and T2: after 12 months). P-value was tested using Mann–Whitney test for non-parametric PAI data. P-value was set to 0.05, *ns* not significant.

**Figure 3:**
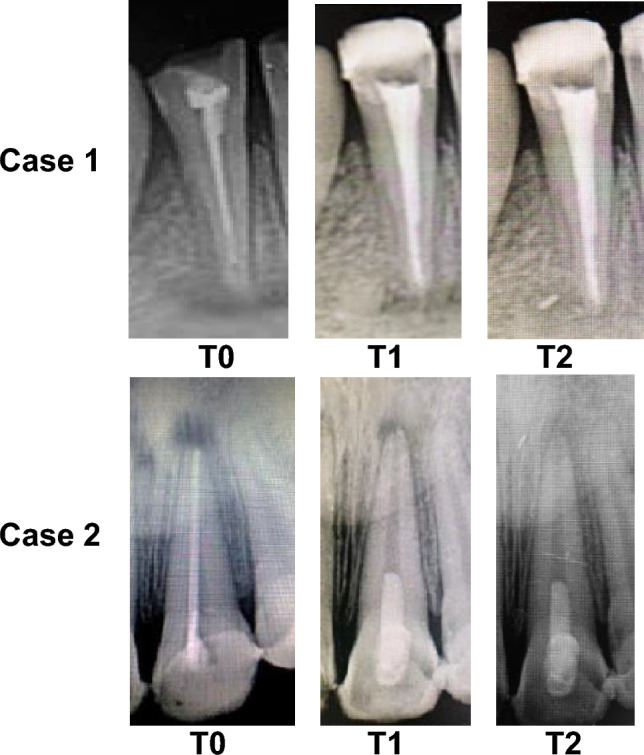
Periapical radiographs of two cases at the follow-up intervals (T0: preoperative, T1: after 6 months, and T2: after 12 months) shows the progress of healing of the periapical radiolucency. Case (1) represented a 13-year-old male. Tooth #41 has retreated with non-surgical endodontic treatment. Case (2) represented an 11½-year-old female. Tooth #21 was treated with REPs. At T1 and T2, both teeth showed radiographic success.

The data in Fig. 2 showed no significant difference in the PAI median scores between the intervention and control groups at T1 and T2. As displayed in Fig. 3, the dimensions of periapical radiolucency decreased in the control and intervention groups over 6, and 12 months, respetively. At 6 months, the PAI medians of the REPs using BC formation and NS-RCR were 1.5mm and 2mm (*p* = 0.108), and at 12 months the PAI medians became 1 mm for the two groups (*p* = 0.274). In the control group, periapical radiographs of all retracted teeth revealed optimal obutration.

The EPT readings of ‘1–39’ indicate vital pulp, ‘40–79’ indicate the non-vital section of the tooth, and ‘80’ indicate a non-vital tooth, according to the manufacturer's guidelines. After 12 months, 18 teeth (54.54%) showed positive readings on the EPT. There was a significant difference between T1 and T2 in terms of pulp sensibility (*p* < 0.0001). The mean difference (T2–T1) was 12.58 with a 95% CI of 8.98; 16.18.

## Discussion

The null hypothesis (*H*_0_) of the primary outcome states that there is no difference in the radiographic diminishing of a periapical radiolucency of treated mature permanent incisors with apical periodontitis between the REPs using BC formation and NS-RCR. The secondary outcomes were to assess the clinical success of both groups and the pulp sensibility of retreated mature permanent teeth with REPs. Based on the results of this study, the null hypothesis (*H*_0_) of the primary and secondary outcomes was accepted.

A concentration of 1.5% of NaOCl was used because its extrusion beyond the apex, particularly in teeth retreated with REPs, is cytotoxic to apical papilla stem cells and inhibits odontoblast development. This concentration was used to strike a balance between the NaOCl's negative and positive effects^27^. However, Fouad et al.^28^ suggested the use of a higher concentration of NaOCl in conjuction with minimal mechanical instrumentation. A side-vented needle was used for irrigation to avoid the forcing of NaOCl solution, debris, and foreign materials into the periapical region. The use of NaOCl and EDTA has demonstrated adequate efficacy in preventing the growth of multiple kinds of mature biofilm^29^. The EDTA neutralises the harmful effect of NaOCl and permits the release of growth factors from the dentine^30^. Transforming growth factor-beta 1 (TGF-β) appears to be released more effectively after EDTA is activated by ultrasound^31^.

The CH intracanal medication was used between appointments because the apical papilla's stem cells are not cytotoxic^32^. The CH reduces the bacterial load, pro-inflammatory cytokines, and matrix metalloproteinases^33^. There is a debate regarding the required duration of CH inside the root canal. Most revitalization protocols consider 1–3 week intervals when the CH has been used as an intermediate root canal dressing^15^.

To allow the influx of mesenchymal stem cells (MSCs) from the periapical region into the root canal space, the apex was manually breached with a K-file size #35 during the second visit^34^. Two previous studies concluded that apical diameters corresponding to size #35 were enough for successful REPs^35,36^. According to the available data, the success of REPs in mature permanent teeth was not significantly impacted by apex diameter^19^. In 2018, Fang et al.^37^ compared the REP success after the use of endodontic files with different cross-sectional diameters. For sizes between 0.5 and 1 mm and 0.5 mm, equivalent REP success proportions of 95.65% and 90%, respectively were reported.

Biodentine was applied over the formed BC to improve cell adhesion and viability over the mineral trioxide aggregate (MTA)^19^. Biodentine prevents coronal tooth discoloration^38^. Additionally, biodentine had adequate biocompatibility, bioactivity, and a strong antibacterial effect. To prevent discoloration, resin adhesive was placed on the walls of the access chamber and the coronal portion of root canals^39^. An in-vitro study assessed the tooth discoloration effect of two calcium silicate-based cements after one year and showed a significant delay in the dicoloration of biodentine compared to ProRoot MTA^40^. The REPs protocol recommended a maximum depth of 3–4 mm of biodentine below the cementoenamel junction into the root canal^41^. An ex-vivo study compared the color discoloration of four biomaterarials used in REPs of extracted premolars. Biodentine showed the best color stability compared to ProRoot MTA, which showed the greatest color variation^41^. Another laboratory study compared both materials (ProRoot MTA^®^ or Biodentine™) in terms of color stability using an acrylic tooth model over 6 months^42^. The outcomes of this study confirmed the significant color stability of biodentine over MTA^42^. Other advantages of biodenine were the ability to apply the restorative material immediately (12 min or 3 min, respectively) after biodintine application and the superior average shear bond strength in comparison to the other two biomaterials^43^. These findings were in agreement with the current study outcomes.

The change in periapical radiolucency is a valid indicator to monitor the bone healing progression and the treatment's success or failure^17^. In several investigations, the periapical radiolucency dimensional changes are assessed using the PAI because of its high inter-examiner agreement compared to other tools such as the Strindberg system and the probability index^44^. The use of the long cone approach with a radiographic stent helped to improve the accuracy of the monitoring and interpretation of dimensional changes.

The results of this study revealed that the two groups had comparable diminishing radiographic radiolucency and comparable clinical success rates during the follow-up periods. The findings of this study found a significant difference in the PAI medians after 6 months, this difference vanished after 12 months. In the REPs groups, one tooth displayed radiographic and clinical symptoms of failure. This was due to the loss of coronal restoration and possible leakage.

Although no previous study tested the efficacy of REPs in the retreatment of mature teeth, the findings of this trial were in line with the high success rates of a few studies that have also focused on the revitalization of mature teeth. After a year of follow-up, Arslan et al.^17^ revealed a success rate of 92.3% for revitalized necrotic incisors. Another study by El-Kateb et al.^19^ compared REPs to BC formation following the use of two different Protaper Next files (PTN) (X3 vs. X5 files). Another prilemenary prospective trial evaluating two REP methods (platelet-rich fibrin versus BC) reported 100% success at 3, 6 and 12 months^6^.

Regarding the NS-RCR, analysis of survival rates from other trials revealed great short-term success, which was consistent with the current findings. For instance, a 7-year retrospective study^45^ included 48 teeth that reported survival rates of > 90% after 12 and 24 months, then declined to 77% after 7 years. The survival rates of teeth treated with three different treatment techniques were evaluated systematically^46^. Over 10 years of follow-up, traditionally retreated teeth had good survival rates, ranging from 84.1 to 88.6%.

Positive responses to the EPT were detected in 54.54% of teeth after 12 months. Previous trials confirmed our conclusion. For instance, 50%, 60%, and 75% of teeth treated with REPs positively responded to EPT after a year of follow-up^6,12,19^. In another study, after a year of treatment with encapsulated human umbilical cord MSCs in a plasma-derived biomaterial for REPs, half of the mature permanent teeth with periapical pulp lesions recovered their pulp sensibility^13^.

A positive response to EPT is not the exclusive indicator of the success of REPs, especially with mature teeth. Radiographic evidence of healing and other signs, such as declining clinical success, may be the only characteristics of REP success in mature teeth^47^. Further investigations are required to cover this point.

The study's uniqueness and approved standardization norms may be its strongest suit. The current study could aid in establishing evidence regarding the use of REPs in the retreatment of mature teeth, especially with the adequate sample size that has been adopted. On the other hand, The main limitations were (1) the short-term follow-up, so prospective clinical trials with longer follow-up recalls are strongly recommended; (2) the radiographic analysis relied on 2D radiographs. Therefore, additional prospective clinical studies using 3D images are beneficial, especially in determining the reduction in the periapical lesion size^48^; and (3) the trial included only mature incisors within certain age group. Therefore, the generalizability of the study oucomes have to be considered carefully.

## Conclusions

In sum, it can be concluded that both the REP and non-surgical retreatment of mature necrotic teeth with periapical radiolucency showed comparabe diminishing of periapical radiolucencies and equivalent clinical results. After a year, the REPs group had recovered up to 56% of their pulp sensibility. REPs could be a good substitute for traditional, NS-RCR.

## Data Availability

The datasets used and/or analysed during the current study available from the corresponding author on reasonable request.
